# Bibliographical Notices

**Published:** 1849-10

**Authors:** 


### BIBLIOGRAPHICAL NOTICES.

*Parturition, and the Principles and Practice of Obstetrics.* By W. TYLER SMITH, M. D., London; Lecturer on Obstetrics in the Hunterian School of Medicine. Philadelphia, Lea & Blanchard, 1849. pp. 395. 1 vol. 12mo.

The English press has of late years teemed with treatises on obstetrics, and their reproduction in the United States has proceeded almost *pari passu.* The evidence of the importance of the subject, and of the interest it has created, derived from this source, is sufficiently evident; we hope as large a profit has been derived by readers from the general diffusion of such publications, as by booksellers.

The position held by obstetrics has for a long time, in the eyes of the intelligent world, been an equivocal one. Nature alone, in most cases, being capable of accomplishing all required, and the act of labor involving feelings of delicacy on the part of the female, a disposition to run the gauntlet, and trust to such assistance as might be at hand from the same sex, has placed the art among the handicrafts, for the skilful performance of which no rules can be given. Or it may be supposed that, like the making of butter, cheese, and such articles, a certain knack being acquired, it belongs entirely to the practiser. We have been amused to hear such ground taken even by eminent medical men, who had grown ancient in their vocation, whose views were those of their youth, tinctured with the prejudices of the early and necessarily rude condition of society in which they were educated. In some countries, and among a people remarkable for simplicity of living, and healthfulness of constitution, such ideas may be entertained, without any danger of diminution to the population, or in the aggregate of great personal suffering. This is not, however, the state of the whole world; and communities exist, so altered in physical constitution from causes which are inherent to civilization itself, that humanity cries aloud for help, and help has come by scrutinizing nature. Upon this is founded science, and although not apparent in its application to every case which occurs, yet cases do occur often enough to keep alive the spirit and the necessity of science for the mitigation of the dangers which attend them. "Obstetrics, the Science and the Art," as a late writer has felicitously been pleased to term it, may well lay claim to importance as a branch of practical medicine, in spite of lampoons on Dr. Slop, or merrymaking at the expense of accoucheurs.

The book whose title is at the head of this article, is an evidence of the original direction which a reflecting, ingenious, cultivated, though perhaps prejudiced, mind may take. It is creditable to the author, and we think will be advantageous to the profession. We have read Dr. Smith's Lectures with pleasure, and shall now proceed to give an account of them.

The form of lectures has been adopted, a form which has not been altered from the mode in which the author's teachings were delivered; on this account there is not noticable the stiffness and precision of more formal publications. Another charm of the work is the absence of an affected nomenclature, which of late years has been carried to such lengths as to be prejudicial. The pedantry of some authors in this department is disgusting; equivocal Greek and Latin transformed into English, is neither instructive nor agreeable, and bad taste alone can dictate it. An idea (for these mongrel names are intended to convey ideas) is rather confused than simplified by such a process. The introductory is devoted to a "comparison between British and Continental obstetric medicine." Preceding this is a vindication of the obstetric art, and an exposition of its character, as chivalric as it is true; every lover and respecter of the sex will accord with the sentiments expressed in the following language: "The excellence of obstetric medicine is one of the most emphatic expressions of that high regard and estimation in which women are always held by civilized races. The state of the obstetric art in any country may be taken as a measure of the respect and value of its people for the female sex; and this in turn may be taken as a tolerably true indication of the standard of its civilization. Schlegel, alluding to the historical growth of nations, calls the institution of marriage the real commencement of civilized life. It may be declared as a truism, that obstetric science must flourish most in countries where the marriage tie is most respected—where women are held in the highest esteem."

One of the causes instrumental in the elevation of obstetrics is the transfer of the practice from the hands of the midwife to the thoroughly educated physician; as expressed by Dr. Smith, "a triumph which has well nigh transferred the allegiance of the lying-in woman from Lucina to Apollo." In England, as it appears, this transfer is not complete, as the signs of the existence of the midwife are occasionally seen in the glowing report of some lying-in charity, the advertised sale of a child's caul, or the newspaper report of a fatal child birth accident. The Royal Maternity of London is conducted by midwives. As far as we are aware, no such institution in the United States is dependant on operatives of this description. An attempt was made by some of the older physicians, in one of the eastern cities, some years since, probably for the purpose of retaining their practice, to introduce a bona fide midwife. It has not however been followed by an increase of the corps, and will not be. Even in instructing nurses, and the establishment of nurse charities, unremitting surveillance is necessary, or such charitable efforts will degenerate into baneful enterprises.

The comparison between British and Continental obstetrics is introduced by the following paragraph. "I think we may take the state of obstetrics in France as the type of continental practice, and I do not think the British school need fear the most severe comparison. Almost all observers concur in the opinion, that throughout the continent, the ties of marriage are less severely observed than in this country. The social organization is less favorable to domestic union and constancy. This is reflected with tolerable accuracy in the general condition of the obstetric art.

We must ever honor the names of Ambrose Paré, Guillemeau, Mauriceau, Portal, La Motte and others of the older French school, as among the great fathers of modern midwifery; but I think it will be granted that cotemporary habits and manners in France are unfavorable to the general cultivation of midwifery, as a branch of the profession claiming equal dignity and usefulness with medicine and surgery. The obstetric art must, I have maintained, flourish most where women are held in the highest esteem. We may admit that the French are a polite people, but they have their paradoxes in matters of feeling as well as in politics. Gentlemen on the other side of the channel most earnestly assist the fold of a shawl, or perform slight services with the greatest gallantry and devotion; yet they consign their women in the hour of travail to the care of their own sex. Such is the fashion and custom of the time." For this exposition we must make a small degree of allowance for national prejudice, and a little more for zeal to clear the face of the earth of that excrescence, as it may be in Great Britain, the midwife. There is no Englishman who does not think his country, with her laws, institutions, customs, manners and habits, the perfection of all that is human, and although the empty sentimentalism of the French may not be pleasing to him when compared with the straight forward, knock down manner of his own countrymen, much might be said by the former in the way of recrimination, especially if the poor rate system and other abuses, together with the vast multitude of degraded and demoralized individuals who throng the streets of London, are marshalled in opposition. A comparison of national morality is a delicate subject, and a beam may, perchance, be in the eye of one who sees a mote in his neighbor's eye. We desire not to pursue this subject further, and will merely say that if avowed illegitimacy in noble quarters be an index of morals, we need go no further than English history to find it.

In summing up the differences between British and Continental practice, the following appear to be the matters which are particularly in relief, the picture of each being presented. For the British —the partiality for craniotomy,—the induction of premature labour, —the proposed separation of the placenta in placenta previa,—the dislike of the Cæsarian section, the Sigaultean operation, and the frequent use of the long forceps. For the Continental—the favorable opinion entertained of the Cæsarian operation—the high opinion in which the Sigaultean operation has been held—the frequent use of the long forceps—the great dislike of craniotomy, and of the induction of premature labor. A somewhat curious mode of explaining the reason of this difference is given by our author—viz., the difference of religious faith professed in England and on the Continent. In fact, the English line of practice has been boldly stated by the author to be *Protestant*, in contradistinction to the other as *Roman Catholic.* We confess we are not convinced by the statements made, either of so great a difference or its cause. Although the Church establishment of England is Protestant, it proceeded from and was originally Roman, the latter faith, moreover, is abundantly spread throughout the kingdom, and we are not aware that when Lutheran or Calvinistic doctrines were introduced, they were attended with a Herodian recklessness of infant life. The option, if it be forced upon the practitioner, between the life of the mother or that of the infant, is inherent in feeling and common sense; the loss of one creates a family void which is irreparable ; the loss of the other is transient in its effects, and comparatively trivial. We do not see that priestly dogmas should more effect the medical mind, than they did that of Napoleon, who throughout his career sacrificed thousands annually of unanointed bodies on the altar of his ambition. The ridicule which was intended to be thrown upon the learned doctors of the Sarbonne by Swift, in his speculations as to the mode by which Tristram Shandy could be baptised, are as applicable to the high church party as to the Catholics; if the "lament of the elder French accoucheurs for the soul of a fœtus lost during delivery" were heard on the one side of the channel, we opine a corresponding groan at the same period was elicited by the same circumstance upon the other. It is a christian principle to save life, both of mother and child, but to hold the scales so as to allow one or the other to preponderate is a reproach to christianity, whether Protestant or Roman Catholic. The French are bold and daring surgeons. Does not this come from their dense population and hospital system ? Who has been bolder than Sir Astley Cooper when he tied the aorta?

In America (North,) the child of England, an objection has suggested itself, that, being Protestant, continental opinions and practices prevailed, and therefore they could not be of religious origin. All sects are tolerated in these United States, and with respect to medical opinion it may be said to be eclectic. The masters and teachers of the present race of practitioners were for the most part educated in the schools of England or of Scotland. As pupils in London or Edinburgh they imbibed decided predilections. Besides, the earlier treatises on the especial branch we are now discusssing were all English. We recollect the time, not long ago too, when Denman, Burns, Smellie and Wm. Hunter were the only authors whose productions could be procured by students. No French obstetrics were taught until the appearance of Baudelocque's treatise, and of the work of Dr. Dewees, based upon it. At the present time the reigning authorities are Churchill, Ramsbotham, Lee, and Rigby, the sales of which are threefold those of Moreau, or Velpeau, or Chailly. If the opinion be entertained by Dr. Smith, "that notwithstanding the blood relation between the United States and this country, American midwifery is far more the child of France than England," we would remark that such deduction from the exposition of any one treatise published in this country, is as little to be relied on as that one swallow makes a summer.

The introductory lecture is remarkable for its boldness, and the strong presentation of the views held by the lecturer; he has assumed and acted upon the license awarded to introductories. The distinctive peculiarity of these lectures, taken as a whole, is the exposition and development of the doctrine of reflex motor action. This doctrine will be found to run through explanations of the physiological movements which acompany the act of conception and parturition. In no system of obstetrics has so much been predicated upon the nervous arrangement by which all the organs, involved in the acts mentioned, are supplied, as in the present. The author is a thorough disciple of Dr. Marshall Hall's views of nervous action, and their application to the reproductive functions has opened a new field for inquiry and investigation. Without following the order of the lectures, we shall endeavour to sketch the ideas and arguments presented to us.

The first point to be established is the existence of nerves. "Sympathies and synergies innumerable between the uterus and other organs, as well as the scalpel of the anatomist, proclaim the existence of uterine nerves, both in the impregnated and unimpregnated states. The nerves of the uterine system can be best studied as a distinct group, and for this purpose they form a class almost as well marked as the respiratory nerves."

They are—1. *Nerves of the ovaria,* derived from the renal and spermatic plexuses. Dr. R. Lee has demonstrated them in some recent dissections in considerable quantity. They exhibit ganglia upon the surface, and often entering the substance of the ovaria. They pass in company with the spermatic artery, and pervade the whole organ. They are distributed by fibres to the fundus uteri, os and cervix.

2. *Nerves to the fallopian tubes.*—These are from the hypogastric regions.

3. *Nerves of the uterus,* from the hypogastric and sacral nerves, and by branches from the spermatic plexus. "Below the bifurcation of the aorta, the aortic plexus divides into two hypogastric nerves. Dr. Lee describes the hypogastric nerve as forming in its descent to the cervix uteri, the hypogastric plexus. This plexus, when it reaches the cervix, terminates in a large ganglion. The hypogastric ganglion is, in the unimpregnated state, on the authority of the same anatomist, from half an inch to three-quarters of an inch in diameter, and is made up of numerous lesser ganglia with their rami of communication. Into the outer and lower surface of the hypogastric ganglion, numerous branches enter from the third, and sometimes from the second and fourth sacral nerves. This ganglion, thus composed and reinforced, is considered by Dr. Lee as the centre from which each lateral half of the uterus is supplied; from the hypogastric ganglia, nerves pass in various directions to the os, cervix, body and fundus, and are distributed extensively to the muscular structure and the internal surfaces of the uterus. In the course of their ramifications over and in the substance of the uterus, numerous ganglionic enlargements occur. In the virgin uterus, Dr. Lee has especially directed attention to a beautiful ganglion in front of the hypogastric ganglion, extensively connected with the surrounding nerves, which he has called the Lawrentian ganglion, in honor of Mr. Lawrence; and in the gravid organ Dr. Lee has described numerous sub-peritoneal ganglions and plexuses on the anterior and posterior surfaces of the organ. These ganglia and plexuses maintain extensive communication with each other and with the hypogastric ganglion below, and the spermatic plexuses and ganglion above. The nerves of the virgin uterus are sinuous or undulating in their course, and invariably accompanied to their termination by small arteries."

4. *Nerves of the vagina,* from the hypogastric ganglion and spinal sacral nerves.

5. *Nerves of the external parts of generation.*—The vulva and perinæum are chiefly supplied by filaments of the genito-crural nerve, branches of the anterior sacral nerves and the perinæal branches of the pudic nerve.

Besides the preceding account of the distribution and arrangement of the nerves supplying the uterus and its appendages, a very important point is presented in the character and derivation of these nerves; we shall adopt the author's exposition of this also. "In the first place the chain of thoracic and abdominal ganglia of the sympathetic communicate with both the sensitive and motor roots of the spinal nerves, and in this way the cerebral and spinal fibres become mingled with the fibres of the sympathetic, and fibres from the sympathetic communicate with the spinal roots. The sympathetic nerves are chiefly formed by these intercommunicating fibres, from the spinal roots to the ganglia of the sympathetic. The great splanchnic nerve arises from the sixth, seventh, eighth, ninth, and tenth thoracic ganglia, and according to Dr. Beck, from the superior thoracic ganglia also. The lesser splanchnic arises from the tenth and eleventh thoracic ganglia. The great splanchnic enters the semilunar ganglion, and it is by the two semilunar ganglia, and their communicating filaments and lesser ganglia, that the solar plexus is chiefly formed. Into the solar plexus some of the terminal filaments of the pneumogastric and phrenic nerves also enter. These fibrillæ from the phrenic and pneumogastric may terminate in the liver and kidney, or they may pass through the superior plexuses of the abdomen, to reach the inferior abdominal, and even the pelvic plexuses. The renal plexus is formed by branches from the solar plexus, and the termination of the lesser splanchnic nerves; the renal plexus also receives fibres from the vagus, and the spermatic plexus is a sub-plexus of the renal. From the spermatic plexus the ovaria and part of the fundus are supplied. The inferior aortic plexus is the last of a series of abdominal plexuses, and it is connected by a chain of ganglia and plexuses with the solar and renal plexuses and with the latter lumbar ganglia. From the inferior aortic plexus the hypogastric nerve descends, forming the hypogastric plexus and the hypogastric ganglion; with this ganglion branches of the sacral nerves unite; and from this nervous estuary the uterus is finally supplied. If we adopt the view, that the plexuses of the abdomen, like the external plexuses, are mechanical adaptations for mixing nervous fibres from different sources, and and apply it to the uterine nerves, it becomes a possibility, and I may say a probability, that the uterine nerves are more variously derived than any other nerves of the body." Again: "The nervous lines I have been enumerating are quite sufficient to account for the communication between the uterus, the brain, and the spinal marrow. The communications between the numerous ganglionic centres and the uterus, are still more obvious, and there is little question but that the great mass of the uterine nerves is composed of ganglionic fibres."

The nerves of the unimpregnated uterus were first described by Wm. Hunter. Tiedemann next described them, and they have since been elaborately examined by Drs. Lee and Beck, who agree with respect to their existence, but differ as regards their size and distribution. Dr. Smith strongly contends for the opinion of Dr. Lee, that the nerves of the gravid uterus are greatly enlarged beyond what they are in the unimpregnated state.

Based upon the truth of this exposition of the nervous supply, and arrangement of the nerves afforded the uterus, is the doctrine of nervi-motor action, or, as it is called, *reflex nervous action* of the uterus. This doctrine in the case of other organs was first promulgated and illustrated by Marshall Hall. It has been applied, carried out, and illustrated with respect to the uterus. All the parturient phenomena are in fact predicated upon it.

The forms of motor action are voluntary, emotional, *excito-motor* or *reflex*, and the peristaltic. The two first and last are accessory. The reflex movements are primary; volition and emotion may be suspended or altered, and the reflex peristaltic movements be unimpeded.

The following paragraphs will explain the author's exposition of reflex action.

"The Reflex actions of the uterus are very numerous, and it is upon these, and the numerous extra-uterine reflex actions excited during the process, that the natural performance of parturition essentially depends. Contraction of the uterus, from irritation of the mammæ, as in the act of suckling the child; contraction of this organ from the cold water douche, applied to the vulva or the abdominal surface; contraction excited by irritating the rectum, as by stimulating enemata; or of the stomach, by drinking a gulp of cold water; of the ovaria, by the presence of the menstrual nisus; of the vagina, by manual irritation, as in "taking a pain;" of the os uteri by irritation, as in the introduction of the hand into the uterus—are all to be considered as so many instances of reflex spinal action. Thus, in parturition, the uterus may be excited, in a reflex form, by irritation of the mammary incident excitor nerves; the pubic and abdominal branches of the intercostals; the rectal; the gastric division of the pneumogastric; the ovarian nerves; and also by the nerves of the vagina, and the os and cervix uteri.

"Many of the different forms of abortion—particularly when the causes are extra-uterine—strikingly illustrate the reflex action of the uterus. A series of cases of abortion would be one of the best expositions of reflex uterine action. Abortion may be caused by irritation of the mammæ, from the sucking of an infant, after milk has ceased to be secreted, as in cases in which the mother becomes pregnant during lactation; abortion may be excited, as a morbid reflex act, from irritation of the bladder, by a calculus; by irritation of the tri-facial nerve, as in cutting the dens sapientiæ; by the mechanical irritation of coïtus; by plugging the vagina; by disease of the os and cervix uteri—malignant or simple induration, inflammation and ulceration; by the irritation of the placenta attached within the uterine mouth; by ovarian irritation in ovarian disease; by irritation of the rectum, as from ascarides, and the use of irritating purgatives or enemata; by puncturing the membranes, and evacuating the liquor amnii, so as to bring the head of the fœtus to act as an excitant to the os uteri; by irritation of the inner surface of the uterus itself, in cases of blighted fœtus, where the ovum acts as a foreign body; by riding on horseback, or any other violent exercise calculated, by succussion, to bring the head of the fœtus into violent contact with the os uteri; and by other sources of irritation to incident spinal nerves which might be enumerated. All these are so many instances of uterine reflex action, the distant parts of the economy being brought into connection with the uterus through the medium of the spinal marrow and its special incident excitor and reflex motor nerves. These facts are of most extensive practical application in devising means for the prevention of abortion.

"In cases of abortion in which the irritation is applied to the os or cervix uteri, or the internal surface of the organ, the immediate action depending on the irritability of the uterus itself is called forth, in connection with true reflex action; but in the instances in which a distal organ is irritated, there can be no doubt whatever of the purely reflex nature of the uterine action which ensues.

"In the lower animals, reflex parturient phenomena are constantly observed; the expulsion of the egg of the common fowl has been caused by sprinkling a few grains of salt upon the vulva or velabrum; cold injections have been used empirically, to excite uterine contractions during foaling or calving, in the mare and cow; and the tipula and libellula have been observed, during the period of oviposition, to deposit eggs, whenever they have been shaken upon rough paper, or when the surface of the abdomen has been irritated in any way: these insects, the silk-worm moth, and others, go on with the work of oviposition even when the abdominal have been divided from the thoracic portions.

"But besides the uterine reflex actions, there is in the Expiratory actions, supervening in the course of labor, from the irritation of the presenting part of the fœtus, another remarkable series of reflex parturient actions, extra-uterine in their seat, but which combine and harmonize in a remarkable way with the reflex actions proper to the uterus. It is found that tumours in the vagina, or the introduction of the hand of the accoucheur, may produce the same result as the pressure of the presenting part of the child in labor. It has been noticed of tumours, which produce little or no motor disturbance while they remain in the cavity of the uterus, that no sooner do they pass from the uterus into the vagina, than "bearing-pains," as they are termed, are produced. In other words, the action of the expiratory muscles is excited in a reflex form by irritation of the vaginal mucous surface.

"In fully-formed labor, then, we have to study the reflex actions of the uterus in combination with the reflex actions of the respiratory muscles, as they are excited in parturition. By these latter reflex actions, the cavities of the thorax and abdomen become involved as auxiliaries to the uterus, and their expulsory actions combine with the proper reflex action of this organ, to effect the expulsion of its contents. But there are also opportunities of observing the reflex action of the uterus singly; such, for instance, as in cases of paraplegia from disease of the middle portion of the spinal marrow. Here the reflex expiratory efforts, the influence of volition and emotion, are entirely withdrawn, it being quite impossible that these actions can be brought into play when the connections of the uterus with the medulla oblongata and the cerebrum, the centres of the respiratory and voluntary actions, are severed. Labor is, in such cases, reduced to very simple elements, being performed solely by the reflex action of the uterus and its peristaltic power.

"In natural labor, after the process has fairly commenced, it is the ovum which furnishes the chief stimulus to the incident excitor nerves, in its transit through the different portions of the parturient canal. Besides the mere enumeration of the various spinal excitors, by the irritation of which the uterus may be affected physiologically or pathologically, we shall have to study the order and succession of the normal reflex actions, uterine and extra-uterine, occurring in labor. Parturition is not one reflex act, but a function, the combined result of many such actions, aided by other powers; and we must study the preliminary phenomena, the different stages of the process, and the final accomplishment of the function; when we shall find that Nature has at her disposal a wonderful succession of stimulus and action, exactly adapted to the dilatation of the os uteri and the vagina; the propulsion and expulsion of the fœtus; and providing, also, for the safe contraction of the uterus, and its return to the unimpregnated state."

We have, endeavoured so far as compatible with our limited space, to give an idea of the leading doctrine of this work. But the whole subject is made to teem with interest, as the articles connected with generation and labour are successively touched upon. The work is a philosophical disquisition rather than a practical treatise. It places before the mind matter for reflection and inquiry, rather than enforces practical dogmas. It may better be entitled the institutes of obstetrics, than a practice of obstetrics, and as such demonstrates that the subject is a scientific one. We recommend the practitioner and student to familiarize himself with and to ponder deeply upon the contents—familiarity with them will make him wise, as experience with such foundation will make him skilful.

*Researches upon the Vital Dynamics of Civil Government.* By Bennet Dowler, M. D., &c. &c. (Reprinted from the New Orleans Medical and Surgical Journal.) New Orleans, 1849. 8vo. pp. 29.

Anything from the intelligent and industrious pen of the author of this pamphlet would be gladly welcomed. His name alone is a sufficient guaranty that the time devoted to the attentive reading of this latest and not the least original of his characteristic efforts will not be thrown away.

The subject he has chosen, needs no argument of ours to establish its importance. It is evidently a favorite one with him, although peculiar and perhaps too unattractive to most medical inquirers. Those who are interested in such studies, will be gratified to find that on this occasion Dr. Dowler has managed to give his theme an aspect which renders it at once as flattering to the national vanity of his professional readers, as it is instructive in the copious amount and variety of statistical information he has brought to bear upon the question. Many a patriotic devotee to less repulsive explorations in the fields of medical investigation, who would shrink from an encounter with such a numerical array upon an ordinary topic, may diligently con the doctor's various tables for the sake of the democratic doctrine they are intended to uphold.

"The facts adduced," we are told at the outset of the argument, "may not prove the doctrine with which they have been associated, but they are not for that reason the less worthy of a careful study; for if they do not justify the suggestions thrown out, they may awaken inquiry, and lead to a more philosophical interpretation." It is further stated, that in the course of his researches, the author has "examined the most authentic works illustrative of vital dynamics, (though that examination may not appear in this paper,) including phrenology, ethnology, psychology, social physics, climatic agencies, the perturbations of immigration and emigration, the geographical distribution of races, the density of population, mortality, marriage, births, the mean duration of life, the physical comforts, as food; also pauperism, industry, capital, education, not to name that all-comprehending subject vaguely denominated civilization." His statement of the object of the essay, and his account of the manner in which he has collected the various data for its basis, are here given in the writer's own words. All must admit the respectability of such credentials; and we need hardly say, they have our hearty commendation.

Thus much for the object of the monograph, and the character of its materials. Next comes the doctrine it proposes to maintain. This may be best presented also in the language of the author—"If vital progression in the Caucasian race be the result of several antecedents, it is probable that the form of government, other things being equal, is the principal element of acceleration, or retardation, according as this government approximates the representative or monarchical principle. Climate, territorial expansion, the mere physical comforts, exemptions from epidemics, immigration, are not the causes of vital movement."

In order to establish his proposition, our author begins by showing (or rather suggesting, for he does not take the time and space entirely to prove it,) that the unexampled progress and improvement of population in the United States has not been due to immigration; "that the ratio of increase has not been greatly influenced from this cause at any decennial period of enumeration."

He reminds us also that our pilgrim fathers settled the least favorable part of the continent, and that they were "preceded more than a century by numerous emigrants, aided by powerful armies and well appointed navies, all of whom were ambitious to conquer the effeminate and almost unresisting nations that possessed the new world, which teemed with all the elements of natural wealth and mineral treasures."

Yet it is notorious that their descendants have far outstripped those of their neighboring competitors for national supremacy, in all that constitutes a nation's greatness. In fact, they have gone on through generation after generation, prospering and to prosper, while their southern and less independent neighbors have been approaching more and more nearly to a premature decay.

"It could be shown," to quote again, "that the Caucasians of North America were not only few in number, poor, unsupported, but were exposed to the ceaseless hostilities of the most courageous savages known to history, whereby more than half the immigrants came to an untimely end; that North America is, I repeat it, naturally inferior in extent, in fertility, in climate, in salubrity to South America. * * * * *

In the magnificent countries of South America, the cattle multiply as the sand on the sea shore, though food is in abundance, while man only is stationary or is declining."

It must occur at once to the reader, that there are many other scarcely less plausible ways of accounting for this striking difference in the rate and character of progress between the people of the respective portions of our continent. Dr. Dowler admits this freely; and whilst he tells us he has not attempted to meet all the objections to his theory, still he offers the facts he has noted, for what they are worth; and in so doing expresses the desire to know whether it is not true that " the monarchical principle (including under that term the hierarchy and the miscalled republicanism of the South,) has retarded the development of population." How the antagonistic principles may act, he does not pretend to say. His wish, for the present, is merely, if possible, to establish the fact.

In the course of the argument, many startling estimates are given in regard to the extremely slow advance, or the actual decline, of several South American States. These estimates afford us much curious information, and are well worthy of consideration, whether they convert us to the doctor's view or not.

Among other things entitled to especial note in passing, our friend makes it appear that " in three centuries, the entire Caucasian race in both Americas, south of the United States, has not equalled numerically that portion of the Union lying west of the Alleghany mountains, settled by the present generation amid the conflicts of prolonged savage wars with the bravest and most sanguinary nations known in all history."

But the researches of the doctor are by no means restricted to the great features of the progress of the New World only. He presents us with many striking comparisons drawn from the more reliable statistics of the European continent. Some of these would be doubtless read with interest by our readers, and it would give us great pleasure to exhibit them, together with a much fuller exposé and discussion of Dr. Dowler's course, both of reasoning and illustration; but the increasing length of the present notice reminds us of other equally important claims upon our space, and admonishes us that we must bring it to a close.

The author's mode of treating the whole subject, rich as it manifestly is in matter for reflection, seems to us much better calculated to arouse inquiry by its suggestive character than to satisfy by its conclusiveness. More than this is not to be expected from a tract of only twenty-nine pages, on a topic that might easily be made to occupy a volume. It is, nevertheless, a matter of regret, that in the haste to put forth his peculiar belief, and stimulate investigation thereupon, he has not spared the time to sustain his hypothesis with a precision and completeness more commensurate with the importance he attaches to its bearing at the present epoch.

We may derive consolation from the hope, however, that he will soon return to the discussion with renewed spirit and ability, as well as opportunity, to pursue it to the end; and we shall look the more earnestly on that account for the appearance of the large work on vital statistics, upon which we understand the learned author has been for some time past employed.

